# Active health behaviors in patients after thyroid cancer surgery: a qualitative study of barriers and facilitators

**DOI:** 10.3389/fonc.2026.1849020

**Published:** 2026-05-29

**Authors:** Qian Dong, Hui Zhang, Ying Zhang, Li Zhang, Qian Wang

**Affiliations:** 1 Department of General Surgery, The Second Hospital of Nanjing, Affiliated to Nanjing University of Chinese Medicine, Nanjing, Jiangsu, China; 2 Department of Intensive Care Unit (ICU), The Second Hospital of Nanjing, Affiliated to Nanjing University of Chinese Medicine, Nanjing, Jiangsu, China; 3 Department of Nursing, The Second Hospital of Nanjing, Affiliated to Nanjing University of Chinese Medicine, Nanjing, Jiangsu, China

**Keywords:** active health behaviors, barriers and facilitators, postoperative thyroid cancer, qualitative research, thyroid cancer

## Abstract

**Aim:**

To identify the factors that promote or hinder active health behaviors in patients following thyroid cancer surgery, and to provide evidence to support the development of relevant interventions for this population.

**Background:**

In recent decades, the incidence of thyroid cancer has surged globally, nearly doubling in some developed countries. China has similarly witnessed an upward trend in thyroid cancer rates. In the management of patients following thyroid cancer surgery, active health behaviors play a pivotal role. Under the current healthcare model, patients’ engagement and self-management is the key to improving patients’ outcomes, reducing healthcare costs, and enhancing public health standards.

**Methods:**

From May 2025 to August 2025, 15 postoperative thyroid cancer patients hospitalized in the Department of Breast and Thyroid Surgery were selected using purposive sampling. Semi-structured interviews were conducted using a phenomenological research approach. Data were analyzed using the Clapazzi’s seven-step qualitative data analysis method.

**Design:**

A qualitative descriptive study.

**Findings:**

Promoting factors for active health behaviors mainly included the establishment of disease cognition and health beliefs, motivation from social support systems, positive psychological adaptation, and convenient healthcare systems. Barriers comprised information confusion and cognitive misconceptions, social role conflicts, obstacles in the medical system and communication, lack of intrinsic motivation, and difficulty in maintaining habits.

**Conclusion:**

Active health behaviors of postoperative thyroid cancer patients are influenced by multiple factors. This study provides evidence-based support for developing targeted strategies—such as psychological intervention, health education programs, and follow-up system optimization, to meet patients’ needs and improve their quality of life.

**Implications for the profession and patient care:**

Healthcare providers should strengthen factors promoting active health behaviors among patients after thyroid cancer surgery and develop targeted support strategies such as psychological interventions and health education programs.

**Impact:**

Through multifaceted support, patients can overcome barriers and transition from passive treatment to active health management, thereby promoting postoperative recovery and enhancing quality of life.

## 1 Introduction

Thyroid cancer is the most common malignant tumor of the endocrine system and the head and neck region (1). In recent decades, the incidence of thyroid cancer has risen rapidly worldwide (2), nearly doubling in some developed countries (1). China has also seen an upward trend in thyroid cancer incidence (3). According to the 2022 Global Cancer Report by the International Agency for Research on Cancer (IARC) (4), The number of new thyroid cancer cases in China has exceeded 460,000, accounting for more than half of the global new cases and 24.3% of the global deaths. Surgery remains the primary treatment for thyroid cancer (5), offering generally favorable overall prognosis and long survival periods (6). However, patients require lifelong follow-up examinations after surgery, making the overall treatment and care process relatively protracted and complex. Throughout this process, patients frequently encounter multiple challenges such as physical, psychological, and social adaptation. In the management of postoperative patients, their active health behaviors play a pivotal role. Postoperative active health behaviors refer to a series of actions in the process of disease management and health promotion, where individuals consciously assume health responsibilities, actively mobilize available resources, exert subjective initiative, and maintain a healthy balance among work, rest, diet, exercise and emotions (7, 8). Under the current healthcare model, patients’ active participation and self-management is the key to improving treatment efficacy, reducing healthcare costs, and enhancing public health standards. Active health behaviors can significantly enhance patients’ quality of life, accelerate recovery, and prevent recurrence (9, 10).

In clinical practice, many patients fail to maintain ideal positive health behaviors after surgery, while some patients deeply recognize the importance of active health behaviors for postoperative recovery and long-term survival, actively follow medical advice and adjust their lifestyles (11). Many patients have limited knowledge about thyroid cancer, and are unclear about the specifics and significance of active health behaviors post-surgery. They may mistakenly believe that completing the surgery alone resolves all concerns, neglecting subsequent self-management and health maintenance. They often face multiple barriers, such as insufficient health awareness, lacking proper understanding and attention of the disease. They have poor self-management skills, and are unable to effectively plan and manage their lives (12). They have excessive psychological pressure and experiencing anxiety and fear due to concerns about disease recurrence with insufficient social support and lacking care and assistance from family, friends, and society (13).

Currently, research on specific factors promoting and hindering active health behaviors among postoperative thyroid cancer patients remains insufficient, with evidence fragmented and incomplete. Therefore, this study delves into the factors promoting and hindering active health behaviors among post-surgical thyroid cancer patients. It aims to provide evidence for clinical nursing staff to develop personalized health education and intervention strategies. Through targeted interventions, it seeks to help patients overcome barriers, enhance their awareness and capacity for active health behaviors, optimize long-term health outcomes, ultimately improve quality of life.

## 2 Background

The concept of active health emphasizes individual participation as the primary driver, achieving disease prevention and health promotion through shaping healthy behaviors and implementing health interventions. For patients who have undergone thyroid cancer surgery, active health behaviors encompass multiple dimensions including medication adherence, maintaining a healthy lifestyle, and psychological adjustment, all of which influence long-term prognosis (11, 14–17).The World Health Organization notes that health-related behaviors exert a bidirectional influence on individual well-being. However, approximately one-third of the global population engages in insufficient physical activity, and chronic diseases account for 74% of global deaths (18).In this context, research on active health behaviors in the medical field has deepened, forming a multi-level research framework ranging from mechanism exploration to technological application. How to effectively guide patients after thyroid cancer surgery to adopt active health behaviors, alleviate psychological distress, and improve quality of life has become a focal issue for clinical healthcare providers and researchers.

Theoretical models of health behaviors provide significant guidance for disease prevention, treatment and rehabilitation, as well as the promotion and maintenance of physical and mental health. In 2024, Jiang et al (19). pioneered the application of the Multi-Theoretical Model of Health Behavior Change in intervention studies for patients with differentiated thyroid cancer. Their aim was to explore the effectiveness of a mobile health intervention program based on the medication therapy management (MTM) theory in this population. Results from a randomized controlled trial published in 2024 further confirmed the positive impact of mobile health interventions grounded in MTM theory on patients with differentiated thyroid cancer (20). The study concluded that MTM-based mobile health interventions significantly improved anxiety levels, reduced fear of cancer recurrence, and enhanced quality of life among these patients.

Existing research confirms that health education, family support, and self-efficacy can promote positive health management behaviors among cancer survivors (21–24). At the same time, factors such as lack of professional knowledge, financial burden, psychological stress, insufficient social support, and neglect of long-term health management may hinder patients from adopting active health behaviors. Most current research focuses on the overall health status or quality of life of thyroid cancer patients, while systematic studies examining both dimensions—promoting factors and barriers to active health behaviors—remain relatively scarce (25–27). Under such circumstances, identifying the key factors influencing postoperative thyroid cancer patients’ engagement in active health behaviors is particularly crucial. This understanding is essential for designing more effective nursing interventions and health management models.

## 3 Methods

### 3.1 Study design

This study employed a descriptive qualitative research design, collecting patients’ perspectives through semi-structured interviews following thyroid surgery. The research adhered to the Core Qualitative Research Reporting Standards (COREQ) guidelines (28).

### 3.2 Participants

This study employed convenience sampling to select participants from a tertiary hospital in Nanjing, China. Inclusion criteria for patients were as follows: 1) Patients meeting the diagnostic criteria for thyroid cancer according to the “Guidelines for Diagnosis and Treatment of Thyroid Nodules and Differentiated Thyroid Cancer (Second Edition) (29)”; 2) Aged 18 to 65 years; 3) Having undergone surgical treatment; 4) Being conscious with no language communication barriers; 5) Understanding their condition and voluntarily participating in this study. Participants were also required to demonstrate a high level of concern for their own health status or an interest in active health behavior research. Exclusion Criteria: 1) Concurrent severe organic lesions in vital organs such as the heart, brain, or lungs; 2) Concurrent tumors in other sites. Ultimately, 15 post-surgical thyroid cancer patients were included in the study.

### 3.3 Data collection

A semi-structured interview guide developed based on the Health Belief Model (HBM) (30) framework was designed, covering topics such as the formation of disease cognition and health beliefs, the motivation of social support systems, and positive psychological adaptation. The interviews were conducted face-to-face in private meeting rooms. No individuals other than the participant and the interviewer were present during the sessions. All participants provided informed consent and were audio-recorded in Mandarin Chinese using a mobile phone recorder. Audio recordings were manually transcribed verbatim within 24 h after each interview. To ensure consistency and accuracy, all interviews were conducted by the first author, who is a female nursing graduate student with systematic training in qualitative research methods and 3 years of clinical experience.

Initially, the researcher contacted eligible participants in person, explained the purpose and content of the study, invited them to participate, and arranged the time and format of the interview. Before the interview, participants provided verbal informed consent for audio recording. Demographic data were collected at the end of the interview. The interview guide was developed based on the HBM frameworks and revised through group discussions and a pretest with two participants.

Interview durations ranged from 20 to 60 min. Data collection and analysis were performed concurrently until no new information emerged, indicating that data saturation had been reached (31). Ultimately, no new codes were generated. The researcher took field notes during interviews, and no repeat interviews were conducted. The transcripts were returned to participants to verify their accuracy.

### 3.4 Data analysis

This study employed Colaizzi’s seven-step analysis method (32) integrated with directed content analysis using NVivo 14.0 software for textual coding (33). The researchers remained open to the emergence of new themes throughout the analytical process. When the transcript content did not fully align with these predefined themes, themes were iteratively developed and refined via team discussions.

The first author acquired an in-depth understanding of the interview transcripts through repeated careful reading to develop a holistic understanding of the participants. Meaningful statements closely related to the research questions were identified and extracted, and semantic units relevant to the study objectives were highlighted and coded. Recurring significant perspectives were coded to form preliminary meaning units. The coding process involved labeling semantic units with descriptive codes that remained faithful to the original text with minimal abstraction or interpretation, thereby minimizing the risk of omitting essential content. Similar codes were then grouped into themes, theme groups, and categories, with logical relationships established. The inherent relationships between themes and participants were described in detail to ensure comprehensive interpretation, followed by an in-depth analysis of the interrelationships among elements. Codes were classified into predefined or new themes based on their similarities and differences. Finally, the findings were returned to the participants for validation to ensure accuracy and reliability. The coding team, consisting of one nurse with a bachelor’s degree and two nursing graduate students, discussed and revised codes through collaborative meetings until consensus was reached. All authors had experience in nursing ethics or qualitative research. The first author translated representative quotations, codes, subthemes, and themes into English.

### 3.5 Trustworthiness

To enhance the trustworthiness of the study, several methodological strategies were adopted. Participants with diverse roles and educational backgrounds were included to enable multi-perspective understanding of the research issue. To ensure credibility, typical interview responses were directly quoted in the results, and feedback was solicited t from collaborating researchers, experts, and participants. All participants confirmed that the findings were consistent with their experiences. Dependability was achieved through transparent team discussions. Data analysis was independently performed by two researchers and cross-checked by a third to ensure reliability and rigor. Furthermore, the interview protocol was reviewed by experts before formal data collection and validated via a small-scale pilot study to verify its appropriateness for the target population. For transferability, the research context, participant selection, data collection, and analysis processes were documented in detail, supported by rich illustrative examples to facilitate reader understanding. The research team maintained ongoing reflexivity regarding their positionalities to enhance objectivity and accuracy.

### 3.6 Ethical considerations

This study has been approved by the Ethics Review Committee of Nanjing Second Hospital (2022-LS-ky011).Informed consent was obtained from all participants. The study strictly followed the principles of the Declaration of Helsinki (34). Participation was voluntary, and participants had the right to withdraw at any time. Data were kept confidential and accessible only to members of the research team. None of the authors had direct involvement in the clinical care of the participants.

## 4 Results

This study included interviews with 15 patients who underwent thyroid cancer surgery. General demographic information was presented in **Table 1**. Four themes of facilitating factors were identified: constructing disease cognition and health beliefs, motivation from social support systems, positive psychological adaptation, and accessible healthcare systems. Four themes of hindering factors were also identified which included information confusion and cognitive misconceptions, social role conflicts, barriers in healthcare systems and communication, and lack of intrinsic motivation coupled with difficulty of sustaining habits (**Tables 2**, **3**).

**Table 1:** Characteristics of the participants.

No.	Gender	Age (years)	Educational level	Operational status	Medical insurance	Surgical procedure
N1	female	44	Bachelor’s	currently employed	Covered by medical insurance	Right thyroid lobectomy and right-sided lymph node dissection
N2	female	33	Bachelor’s	currently employed	Covered by medical insurance	Right thyroid lobectomy and right-sided lymph node dissection
N3	female	26	Bachelor’s	currently employed	Covered by medical insurance	Total thyroidectomy and left-sided lymph node dissection
N4	Male	42	Senior high school	currently employed	Covered by medical insurance	Left thyroid lobectomy with left-sided lymph node dissection
N5	female	37	Bachelor’s	currently employed	Covered by medical insurance	Total thyroidectomy with bilateral central zone lymph node dissection
N6	Male	33	Bachelor’s	currently employed	Covered by medical insurance	Left thyroid lobectomy with left-sided lymph node dissection
N7	Male	46	Bachelor’s	currently employed	Covered by medical insurance	Right thyroid lobectomy and right-sided lymph node dissection
N8	Male	46	Senior high school	currently employed	Covered by medical insurance	Right thyroid lobectomy and right-sided lymph node dissection
N9	female	34	Bachelor’s	currently employed	Covered by medical insurance	Left thyroid lobectomy with left-sided lymph node dissection
N10	female	58	Junior High School	retirement	Covered by medical insurance	Total thyroidectomy with right-sided lymph node dissection
N11	female	44	Senior high school	currently employed	Covered by medical insurance	Left thyroid lobectomy with left-sided lymph node dissection
N12	female	38	Bachelor’s	currently employed	Covered by medical insurance	Right thyroid lobectomy and right-sided lymph node dissection
N13	female	37	Bachelor’s	currently employed	Covered by medical insurance	Total thyroidectomy and left-sided lymph node dissection
N14	Male	46	Senior high school	currently employed	Covered by medical insurance	Right thyroid lobectomy and right-sided lymph node dissection
N15	female	53	Senior high school	retirement	Covered by medical insurance	Right thyroid lobectomy and right-sided lymph node dissection

**Table 2:** Teme summary and representative participant responses.

Theme	Representative participant responses
Building disease awareness and health beliefs	N1: “When I first heard the word “cancer,” it felt like the sky had fallen. I thought my life was over. But the doctor told me that thyroid cancer is more like a chronic condition. With proper management, it can be lived with long-term, just like high blood pressure or diabetes.”N4: “Taking Euthyrox every morning has become as routine for me as brushing my teeth and washing my face.”N7: “I still feel a bit nervous before each follow-up appointment. Seeing “no abnormalities” on the ultrasound report and all blood test results within normal ranges reassures me that these months of persistence haven’t been in vain.”N11: “I feel that whether this illness can be kept under control largely depends on me. Taking medication on time, eating healthily, and maintaining a positive mindset—I feel I’m quite disciplined.”N14: “This illness has taught me how to take better care of myself. I can now read lab reports and know how to plan three balanced meals a day.”
Incentives for social support systems	N2: “If it weren’t for my family’s thoughtfulness, I might have forgotten to take my medicine. They remind me every day at the right time, cook meals specially tailored to my taste, and eat light meals with me.”N6: “When I first got diagnosed, I felt like the sky was falling. But then I learned a friend had the same disease too. Seeing her share how she adjusted her medication and managed side effects really helped me feel more at ease.”N10: “The doctors and nurses never rush me through appointments. They explain everything clearly whenever I ask questions and proactively inquire about how I’ve been feeling lately.”
Positive psychological adjustment	N3: “Before falling ill, I always believed my body was ‘endless.’ Work was hectic, social engagements piled up, and pulling all-nighters became routine. It wasn’t until the moment of diagnosis—as if someone had suddenly pressed pause—that I realized my body had been sending me signals all along, signals I’d consistently ignored. This illness felt like life itself sounding a harsh wake-up call, jolting me out of my numbness.”N7: “I used to chase grades and recognition, but now I seek peace and authenticity. I still work hard, but no longer at the expense of my health. Cancer is like a stern teacher—it didn’t make me conquer anything, but taught me how to coexist with vulnerability and treat myself with greater kindness.”N9: “What truly matters to me? Now I spend my time with family and writing my recovery journal. The pain hasn’t vanished, but it has become a window through which I understand others’ suffering.”
Convenient healthcare system	N8: “No more running back and forth for follow-up appointments—just book them on your phone. Test results get sent straight to me, saving tons of time and effort.”N10: “I used to worry about forgetting to take my medicine, but now my phone buzzes to remind me what to do—it’s like having a little health assistant right by my side.”
Information fog and cognitive misconceptions	N2: “Now that I’ve got this illness, I spend all day searching online. The more I look, the more confused I get—some say I should eat more kelp to boost my iodine, while others claim I shouldn’t touch any iodine at all.”N6: “I feel incredibly stressed about eating right now. Every single bite makes me wonder: Is this okay to eat? Could it be a trigger food? I end up spending half an hour checking my phone before I can take a bite.” N12: “I’ve bought a whole bunch of health supplements—bottles and jars of them. Some claim to boost immunity, others to fight tumors. I’ve spent a lot of money, but I have absolutely no idea if they work. I’m always worried that taking the wrong ones might actually do more harm than good.”
Social role conflict	N1: “Doctor, I’ve been incredibly busy at work lately, constantly traveling for business. When things get hectic or I’m on the road, I often forget to take my medication—sometimes I even forget to bring it along. Follow-up appointments always clash with work commitments. Even when I manage to schedule one, a last-minute trip or meeting forces me to reschedule. I’m frustrated myself, but there’s really nothing I can do about it.”N3: “My children need my care at home, and I spend every day tending to them, constantly putting my own affairs on hold. Whenever they fall ill or something comes up, I always prioritize them first—delaying my own medication or follow-up appointments. It feels like I’ve grown accustomed to this rhythm. I always feel their health is more important and urgent than my own.”N12: “The most frustrating thing about eating out is that I have to go along with whatever others order—I can’t strictly stick to my own dietary requirements.”N13: “At friends’ gatherings, the table is always laden with greasy, salty dishes. When others urge you to eat, refusing makes you seem like a killjoy, yet eating leaves you feeling guilty.”
Barriers in healthcare systems and communication	N5: “For the follow-up appointment, I arrived at the hospital before dawn to queue up. After waiting four or five hours, the doctor only spent a few minutes with me. I ended up wasting most of the day at the hospital.”N7: “Getting an appointment for a follow-up checkup is harder than snagging train tickets during the Spring Festival travel rush! Every day, I wait by my phone at the exact time appointments open, but they’re gone within seconds—impossible to grab. For us out-of-towners, it’s even more of a hassle. We have to take a day or two off work just for the follow-up, plus figure out our own transportation and lodging. The cost of these checkups is just too high.”N9: “Every follow-up appointment feels like a battle—queuing for registration, queuing to pay, queuing for tests, and queuing again to pick up medication.”N11: “I hadn’t even exchanged two sentences with the doctor before they started writing prescriptions. I still had so many questions to ask, but seeing the long line of people waiting behind me, I felt awkward asking more.”
Lack of intrinsic motivation and difficulty sustaining habits	N1: “At first I was super motivated, determined to get my health back on track this time. I followed the doctor’s instructions to the letter, like a completely different person. But honestly, once the novelty wore off and time went on, I … gradually let myself go.”N4: “I made a plan to walk ten thousand steps every day. The first couple of weeks felt pretty rewarding, but then work got busy, or I’d skip it on rainy days to take it easy, and before I knew it, I’d stopped altogether. By the time I remembered, it just didn’t seem worth it anymore.”N6: “Why do you think I’m so persistent? I take my blood pressure meds, I eat light meals, but my body still feels the same. Sometimes I even think those who don’t pay as much attention to their health are happier.”N10: “My family sometimes says, “You started off with such passion, but now you’re back to your old ways.” I’m frustrated with myself too, but I just can’t recapture that initial drive. It feels like I can’t see a clear payoff anymore.”

**Table 3:** Classification of factors and related strategies.

Category	Theme		Strategy
Physical	Hindering factors	Lack of intrinsic motivation coupled with difficulty of sustaining habits	In clinical practice, personalized rehabilitation exercise programs should be developed. According to each patient’s physical condition and interests, with regular evaluation and modification. Patients are encouraged to incorporate journaling into daily life, such as recording rehabilitation exercise times and dietary details, to facilitate self-monitoring. Additionally, establishing patient support groups where individuals can encourage one another and share experiences can enhance persistence and self-management capabilities.
Psychological	Facilitating factors	1. Constructing disease cognition and health beliefs	1. When patients develop a clear understanding of their condition, grasp the key points of postoperative recovery, and recognize the long-term benefits of health behaviors, they will develop an intrinsic motivation to actively engage in such behaviors. 2. once patients deeply understand the importance of active health management—such as regular follow-ups, balanced nutrition, and moderate exercise—in preventing recurrence and promoting recovery, they will actively take corresponding actions, thereby driving their long-term adherence to effective health management.
		2. Positive psychological adaptation	
Social adaptation	Facilitating factors	1. Motivation from social support systems	1. The care, encouragement, and companionship from family, friends, and fellow patients can strengthen patients’ will to survive and motivation to recover, encouraging them to actively engage in treatment and improve their quality of life. 2. Therefore, healthcare professionals should reinforce patients’ commitment to proactive health management and ensure timely and effective communication, jointly facilitating the shift from “passive treatment” to “proactive health.”
		2. Accessible healthcare systems	
	Hindering factors	1. Information confusion and cognitive misconceptions	1. It is recommended to conduct comprehensive information education for patients in the early postoperative period. Healthcare professionals should thoroughly explain disease-related knowledge, follow-up treatment plans, and rehabilitation precautions, while developing personalized, easy-to-understand informational brochures. 2. Consequently, there is an urgent need to provide comprehensive social support resources through multiple channels. In the future, healthcare providers should collaborate with communities and employers to develop personalized social support plans tailored to the specific needs of patients across different age groups, helping them smoothly navigate transitions in their social roles. 3. Regarding doctor-patient communication, healthcare providers should enhance their communication skills, explain diseases and treatment plans using plain language, encourage patients to ask questions, and provide patient answers.
		2. Social role conflicts	
		3. Barriers in healthcare systems and communication	

### 4.1 Building disease cognition and health beliefs

Thyroid cancer patients can understand the “chronic disease” nature of thyroid cancer and recognize the importance of taking medication and undergoing follow-up examinations after surgery. Believing in their own actions—such as taking medication regularly and maintaining a healthy diet—can effectively control the condition and prevent recurrence, thereby gaining a strong sense of self-efficacy. N1: “When I first heard the word ‘cancer,’ I felt like the sky had fallen. I thought my life was over. But the doctor explained that thyroid cancer is more like a ‘chronic condition’—managed properly, it can coexist long-term like hypertension or diabetes.” N4: “Taking Euthyrox every morning has become as routine as brushing my teeth or washing my face.” N7: “I still get a little nervous before each follow-up, but seeing ‘no abnormalities’ on the ultrasound report and normal blood test results makes me feel all these months of persistence haven’t been wasted.” N11: “I feel whether this disease stays stable is largely in my own hands. Taking medication on time, eating healthily, and maintaining a positive mindset—I feel I’m quite disciplined.” N14: “This illness taught me how to take better care of myself. Plus, I can now read lab reports and know how to plan my three daily meals.”

### 4.2 Incentives for social support systems

Reminding patients to take medication, providing emotional support, and helping adjust dietary habits by family and friends serve as direct motivators for patients to persevere. Peer sharing also offers valuable experience, emotional resonance, and role model inspiration. N2: “If it weren’t for my family’s thoughtfulness, I might have forgotten to take my medication. They remind me daily at the right time, cook meals tailored to my preferences, and eat light meals with me.”N6: “When I was first diagnosed, I felt like my world was collapsing, but then I learned a friend also had this disease. Her sharing about how she adjusted her medication and managed side effects made me feel much more at ease.” N10: “The doctors and nurses never brush me off. They explain everything clearly whenever I ask questions and proactively check in on how I’m feeling lately.”

### 4.3 Positive psychological adjustment

Viewing the cancer experience as a “wake-up call” or turning point in life, thereby inspiring positive lifestyle changes (such as starting regular exercise or learning about health). Internalizing healthy behaviors as part of a new way of life. N3: “Before getting sick, I always thought my body was ‘endless.’ Work was hectic, social engagements piled up, and pulling all-nighters became routine. It wasn’t until the diagnosis—like someone suddenly hitting pause—that I realized my body had been sending signals all along, but I never listened. This illness was like life itself sounding a harsh alarm, jolting me awake from my numbness.” N7: “I used to chase achievements and recognition. Now I seek peace and authenticity. I still work hard, but no longer at the expense of my health. Cancer is like a stern teacher—it didn’t make me ‘conquer’ anything, but taught me how to coexist with vulnerability and treat myself with greater kindness.” N9: “What truly matters to me? Now I spend my time with my family and writing my recovery journal. The pain hasn’t vanished, but it has become a window through which I understand others’ suffering.”

### 4.4 Convenient healthcare system

The streamlined follow-up process and comprehensive monitoring system—supported by clear medication logs and scheduled follow-ups—render health management practical and trackable. Guidance from healthcare providers, positive feedback, and ongoing care significantly enhance patients’ trust and adherence.N8: “No more running back and forth for appointments—I can book them right on my phone, and test results get sent straight to me. Saves so much time and effort.” N10: “I used to worry about missing doses, but now my phone buzzes to remind me—it’s like having a personal health assistant by my side.”

### 4.5 Information fog and cognitive misconceptions

Information overload and conflicting advice: Receiving contradictory information from the internet and others, especially regarding “iodine-rich diets” and “health supplements,” can lead to confusion and anxiety among patients. N2: “Since getting this illness, I’ve been searching online every day. The more I look, the more confused I get—some say to eat more kelp for iodine, while others claim you can’t touch any iodine at all.” “ N6: “Eating has become incredibly stressful. Every bite makes me wonder: Is this okay to eat? Could it be a trigger food? I spend half an hour checking my phone before eating anything.” N12: “I’ve bought stacks of health supplements—some claim to boost immunity, others to fight tumors. I’ve spent a fortune, but I feel completely uncertain. I’m always afraid taking the wrong thing might actually make things worse.”

### 4.6 Social role conflict

Conflicts between work and medication management: Busy schedules and business trips lead to missed doses and difficulty keeping follow-up appointments. Pressure from family responsibilities: As caregivers, they often prioritize family needs over their own health management. Social awkwardness: Dining out makes strict dietary control difficult, and carrying medication causes inconvenience. N1: “Doctor, I’ve been incredibly busy at work lately with frequent business trips. When things get hectic or I’m traveling, I forget to take my medication—sometimes I even forget to bring it. Follow-up appointments always clash with work. I finally schedule one, only to have to reschedule due to last-minute travel or meetings. I feel frustrated myself, but there’s nothing I can do.” N3: “My kids need my care. I revolve around them daily, always putting my own needs on hold. Whenever they get sick or something comes up, I prioritize them—taking my own meds later, postponing check-ups. I’ve kind of ‘gotten used to it.’ Their health just feels more important and urgent than mine.” “N12: “Eating out is the biggest headache. I have to eat whatever others order, making it impossible to strictly follow my dietary requirements.” N13: “At friend gatherings, the table is full of high-fat, high-salt foods. If others urge you to eat and you refuse, you seem like a party pooper. But if you do eat, you feel guilty.”

### 4.7 Barriers in healthcare systems and communication

Post-thyroid cancer surgery patients face difficulties scheduling follow-up appointments and endure lengthy queues, which can dampen their motivation. Inadequate doctor-patient communication and brief clinic visits fail to adequately address patients’ psychological concerns and subtle symptom changes, further fueling doubts and distrust. N5: “For my follow-up, I queued at the hospital before dawn and waited four or five hours. The doctor only spent a few minutes with me, spending most of the day at the hospital.” N7: “Getting an appointment for a follow-up is harder than snagging train tickets during Spring Festival travel rush! I wait by my phone at the exact time every day, but the slots disappear in seconds—I can never get one. It’s even tougher for us out-of-towners. We have to take a day or two off work for the checkup, arrange our own travel and lodging—the cost is just too high.” N9: “Every follow-up feels like a battle—queuing for registration, queuing to pay, queuing for tests, queuing to pick up medication…” N11: “Before I could even get a word in, the doctor started writing prescriptions. I still had so many questions, but seeing the long line behind me, I felt awkward asking more.”

### 4.8 Lack of intrinsic motivation and difficulty sustaining habits

At first, patients have the motivation to change, but over time, it becomes difficult to turn healthy behaviors into habits. The lack of immediate positive feedback leads to a decline in patients’ motivation for proactive health behaviors. N1: “I was super motivated at the start, determined to finally get my health in order. I followed everything the doctor said, like a completely different person. But honestly, once the novelty wore off and days turned into weeks, I … gradually slacked off.” N4: “I set a goal to walk 10,000 steps daily. The first two weeks felt rewarding, but then work got busy, or I skipped it on rainy days. Slowly, it fell by the wayside. By the time I remembered, it just seemed pointless.” N6: “Why am I even sticking with this? I take my blood pressure meds, eat lighter meals, but my body still feels the same. Sometimes I even think those who pay less attention to their health are happier.” N10: “My family sometimes says, ‘You started out so enthusiastically, but now you’re back to your old ways.’ I’m frustrated too, but I just can’t recapture that initial momentum. It feels like I can’t see clear rewards anymore.”

## 5 Discussion

### 5.1 Factors promoting healthy behaviors in patients with postoperative thyroid cancer

Numerous factors promote active health behaviors in thyroid cancer patients after surgery, including disease awareness and health belief formation, motivation from social support systems, positive psychological adaptation, and accessible healthcare systems. Research indicated that disease awareness and health belief formation constitute a crucial foundation for thyroid cancer patients to adopt active health behaviors. When patients develop a clear understanding of their condition, grasp the key points of postoperative recovery, and recognize the long-term benefits of health behaviors, they will develop an intrinsic motivation to actively engage in such behaviors. For instance, once patients deeply understand the importance of active health management—such as regular follow-ups, balanced nutrition, and moderate exercise—in preventing recurrence and promoting recovery, they will actively take corresponding actions, thereby driving their long-term adherence to effective health management. Therefore, healthcare providers should employ diverse health guidance strategies to fully stimulate patients’ motivation, evoke positive emotions, and help them develop habits of actively managing their own health. Additionally, the buffering and motivational role of social support systems cannot be overlooked. The care, encouragement, and companionship from family, friends, and fellow patients can strengthen patients’ will to survive and motivation to recover, encouraging them to actively engage in treatment, effectively manage their condition, and improve their quality of life (35–37). Positive psychological adjustment also enables patients to face illness with optimism, viewing post-operative life as a new beginning and proactively exploring healthy lifestyles tailored to their individual needs (38, 39). A convenient healthcare system provides patients with easy access to medical services, enabling them to clearly understand self-management strategies and further promote the development of proactive health behaviors (40). Therefore, healthcare professionals should reinforce patients’ commitment to proactive health management and ensure timely and effective communication, jointly facilitating the shift from “passive treatment” to “proactive health.”

### 5.2 Barriers to healthy behaviors among patients with postoperative thyroid cancer

Patients who have undergone thyroid cancer surgery also face numerous barriers to engaging in active health behaviors. ①Information confusion and cognitive misconceptions: Patients are exposed to a vast amount of information concerning disease recurrence, follow-up care, dietary restrictions, and other related topics. Given the diversity of these information sources, patients often struggle to evaluate its accuracy. Misleading information may cause excessive anxiety, severely impacting recovery progress and quality of life. Therefore, timely and accurate provision of evidence-based information to correct misconceptions is crucial. It is recommended to conduct comprehensive information education for patients in the early postoperative period. Healthcare professionals should thoroughly explain disease-related knowledge, follow-up treatment plans, and rehabilitation precautions, while developing personalized, easy-to-understand informational brochures. Additionally, patients with varying levels of education may differ in their ability to comprehend and accept information, potentially hindering their capacity to make sound health decisions (41). Therefore, healthcare professionals can enhance patients’ understanding and retention using illustrated materials and explanatory videos. Simultaneously, authoritative information should be disseminated via hospital websites and WeChat official accounts for patients to access at any time. ② Thyroid cancer surgery may impact patients’ physical appearance and work capacity, causing maladjustment in social roles such as family and workplace. For instance, some patients face career transition challenges due to physical limitations preventing them from continuing their original jobs. Within families, altered self-identity may lead to conflicts with relatives. Employers should offer greater understanding and accommodation, providing necessary work adjustments and support. Consequently, there is an urgent need to provide comprehensive social support resources through multiple channels. In the future, healthcare providers should collaborate with communities and employers to develop personalized social support plans tailored to the specific needs of patients across different age groups, helping them smoothly navigate transitions in their social roles. ③Ineffective communication of medical information and inadequate doctor-patient communication impede the rehabilitation management of patients after thyroid cancer surgery. Regarding doctor-patient communication, healthcare providers should enhance their communication skills, explain diseases and treatment plans using plain language, encourage patients to ask questions, and provide patient answers (42). ④Most thyroid cancer patients experience a lack of intrinsic motivation post-surgery. Recovery is a long-term process requiring patients to develop healthy habits and adhere to rehabilitation exercises. However, many struggle to maintain these practices over time due to factors such as insufficient disease awareness and low self-efficacy (43). In clinical practice, personalized rehabilitation exercise programs should be developed. According to each patient’s physical condition and interests, with regular evaluation and modification. Patients are encouraged to incorporate journaling into daily life, such as recording rehabilitation exercise times and dietary details, to facilitate self-monitoring. Additionally, establishing patient support groups where individuals can encourage one another and share experiences can enhance persistence and self-management capabilities.

Given limited awareness of the disease, misconceptions about medical information, and internal barriers within the healthcare system, self-care by patients following thyroid cancer surgery can only achieve its intended goals effectively with the support of professional healthcare providers and a robust social safety net.

## 6 Conclusion

This study systematically analyzed the factors promoting and hindering active health behaviors among patients after thyroid cancer surgery. It suggested that healthcare providers should strengthen the promotion of active health behaviors among these patients, such as offering personalized health guidance. Simultaneously, further exploration is needed into pathways to overcome barriers, including how to optimize medical information delivery and reduce information confusion. Only through multifaceted support can patients overcome obstacles, transition from passive treatment acceptance to active health management, and thereby enhance postoperative recovery and quality of life.

## Data Availability

The raw data supporting the conclusions of this article will be made available by the authors, without undue reservation.
